# School-based violence prevention strategy: a pilot evaluation

**DOI:** 10.5249/jivr.v7i2.565

**Published:** 2015-07

**Authors:** Rachel V. Thakore, Jordan C. Apfeld, Ronald K. Johnson, Vasanth Sathiyakumar, A. Alex Jahangir, Manish K. Sethi

**Affiliations:** ^a^The Vanderbilt Orthopaedic Institute, Center for Health Policy, Vanderbilt University, Nashville, TN, USA.

**Keywords:** Violence, Adolescent, Schools, Prevention and control

## Abstract

**Background:**

Violence has recently been reported among a primarily young, minority population in Nashville, Tennessee. School-based programs have been proven as effective methods of reducing violent behavior, beliefs, and actions that lead to violence among adolescents.

**Methods:**

Investigators implemented a rigorous search for an appropriate school-based violence prevention program for Metropolitan Nashville middle school students utilizing a systematic review and discussion group with victims of violence. 27 programs nation-wide were reviewed and 2 discussion groups with African American males under the age of 25 admitted to a level 1 trauma center for assault-related injuries were conducted. Our findings led to a single, evidence-based conflict resolution program. In conjunction with educators, we evaluated the program’s effectiveness in a pilot study in a Nashville middle school with high rates of violence.

**Results:**

122 students completed the conflict resolution program and described their behavior and experiences with violence in a pre-test/post-test self-rate questionnaire. Results showed a significant decrease in violent behavior and an increase in students’ competencies to deal with violence (p less than 0.05).

**Conclusions:**

This study shows that a reduction in violent behavior and beliefs among middle school students can be achieved through the implementation of a targeted violence intervention program. A larger-scale intervention is needed to develop more conclusive evidence of effectiveness.

## Introduction

Youth violence and aggression has remained a major public health issue in the United States in the last decade.^1^ In a national survey of students, 32.8% of adolescents in grades 9-12 reported being involved in a physical fight in the last twelve months, while 12% reported being involved in fights on school premises. Overall, 707,212 people aged 10 to 24 were treated in the emergency departments due to injuries from physical assaults.^2^

Numerous school-based interventions have been developed to reduce violence and associated behaviors, including bullying and aggression, among adolescents. These programs are based on the theory that violent behavior is learned and therefore can be targeted at a young age through appropriate interventions. ^3,4^ Several systematic reviews of school-based violence prevention programs have demonstrated the effectiveness of these programs at reducing youth violence, bullying, and aggression for a variety of age levels.^5-13^ Middle school students, in particular, have been shown to be a highly effective target population for violence prevention curriculums. In fact, Kellam et al. have shown that aggressive behavior in middle school children not only begins as early as first grade, but that it is preventable through early interventions.^3^

However, there has also been contrary evidence demonstrating that specific school-based programs have not been effective at reducing youth bullying or violence.^14-16^ In a systematic review of various types of school-based interventions, Vreeman et al. found that less than half of curriculum-based interventions succeeded in reducing bullying, aggressive behavior, and victimization. ^14^

With an abundance of school-based interventions to select from, not all of which are equivalently successful, a rigorous methodology in program selection is imperative. Several investigators have described the importance in organized development and implementation of a school-based violence prevention prior to research and conduction of a pilot evaluation.^17-24^ The majority of these studies describe the importance of selecting and implementing evidence-based interventions with consideration of the contextual and sociocultural characteristics of the target population along with ongoing collaboration with educators. 

The purpose of our study was to utilize a multi-step approach to select and adopt a school-based violence prevention intervention for middle school youth within Nashville, Tennessee. Metropolitan Nashville is an urban setting with a large low-income, minority population. Similarly to other large, urban centers,^25-27^ high rates of violence have been shown to exist among youth in Nashville, Tennessee.^28^ We hypothesized that a school-violence prevention intervention that is selected in consideration of the context of youth violence among our target population would be effective at reducing behaviors and beliefs related to violence, victimization, and aggressive behavior among middle school children.

Our study consisted of four aims designed to select an effective school-based intervention program and conduct a pilot evaluation in a middle school in Nashville, Tennessee. The first arm of our study consisted of a national review of school-based prevention programs and interviews with program developers. The second component involved discussion groups with young adult, African-American males who were injured in acts of youth violence during adolescence. The third component included collaborating with school leaders and selecting a pilot school for the intervention. Lastly, the intervention was introduced to students at the pilot school and evaluated for its effectiveness using a pre-test/post-test questionnaire.

## Methods

**National Review of Violence Prevention Programs **

Institutional Review Board (IRB) approval was obtained from the Vanderbilt University IRB committee prior to beginning all research activities. The first component of this study was to conduct a program review of established school-based interventions. This search was national in scope, with the primary focus on programs that specifically target violence in the youth community. Programs were located through searches on the Internet, Lexis/Nexus, national literature searches, and bibliographic and program references. As programs were identified, they were entered into a secure Excel database, along with defining characteristics and whether they met our criteria (included below), which were developed and modified throughout the course of the search. Particular focus was given to classroom-based and school-affiliated programs.

 Primary criteria for the review included programs that were as follows: school-based; universal in approach; evidence-based; focused on violence prevention, conflict resolution, and social competency development; aimed at the appropriate target ages/grades (middle school students at-risk for violence); of appropriate duration and scope (within one semester for middle school students) ; affordable (in terms of money, instruction time, and staff resources); offering accessible lesson plans and materials; and offering extensive training and expansion options for long-term use. Once school-based intervention programs were identified, 26 school-based/school-affiliated programs with a comparable target population and similar goals to our proposed intervention were selected for further evaluation and analysis. 19 of those programs were found to still be active with a school-based approach. 

All 19 selected programs were contacted in the summer of 2011 via telephone, electronic mail, and if necessary, US mail, and a telephone interview with the program director or administrator was scheduled. Interviews with administrators of these key programs were then conducted. Each program administrator was asked about different aspects of his or her intervention program. Conversations with each administrator lasting approximately one hour explored several issues including: 1) the program goals and methods 2) the target group 3) risk factors targeted 4) evaluation of program success 5) evidence of results 6) evaluation of long term effect of the program 7) lessons learned and 8) changes that the program directors should make in the development of a future intervention program.

**Discussion Groups **

The next component of our study was a moderated discussion group with young adults in the Nashville community who were injured in incidents of youth violence as adolescents. Demographics for our participants were selected based on a previously published study of 343,866 patients who were admitted to the emergency department of a level I trauma center in Nashville, TN. In this study, Moore et al. reported that black males, especially between the ages of 18-25, were more likely to suffer assault-related injuries than their non-black counterparts (p<0.001).^28^ Based on these findings, patients between the ages of 18 to 25 admitted to a level I trauma center from 2004 to 2010 for assault-related injuries were identified through the hospital’s electronic medical records system. Potential participants were contacted via telephone, electronic mail, or US mail. Participants with histories of youth violence and aggression, including gang affiliation, who were currently involved in violence prevention with at-risk youth were included in our discussion.

Two discussion groups of eight participants each were moderated by a Youth Prevention Specialist at a Youth Center that offers counseling and support to at-risk adolescents in Nashville, Tennessee. Audio recordings of the discussion groups were transcribed, analyzed, and kept for future reference. The discussion groups aimed to ascertain risk factors in the victim’s past that potentially increased the patient’s susceptibility to violence, as well as to determine the victim’s outlook on school-based intervention programs and potential additions to future programs that would make a school based intervention model more powerful. 

Analysis of the two discussion groups led to several key findings. Participants cited a lack of positive, adult role models, including parents, as a pretext for becoming involved in gangs and youth violence. Another key factor behind violence included negative cultural influences, including violent lyrics in songs. Participants described violence and aggression as the only legitimate tools for gaining respect in their socioeconomic context. Gang participation was described as leading to social support and acceptance, a recreational sport, method of protection, and providing access to financial resources. 

Regarding intervention strategies, participants believed programs that focus primarily on teacher instruction would not be effective due to a lack of respect for authority. They believed participation from peers would be an essential component of a violence prevention program. It was also considered necessary for youth to be provided with alternative interests and hobbies, and give them “something to do [other than be out on the streets].” Ideas for interventions included helping youth find options for future career paths and partnerships with community organizations that can provide alternative recreational activities for at-risk youth.

**Program Selection**

The third component of our study involved selecting a single intervention and collaborating with educators in the Metropolitan Nashville Public School system for implementation at a pilot school. Information from the discussion groups, interviews with program directors, and further review of all programs was used to choose 4 universal, classroom-centered, evidence-based programs. One of these, a middle school program called “Aggressors, Victims, and Bystanders” (AVB),^29^ was identified as most appropriate in each of the above mentioned criteria categories.

AVB was developed by educational scientist Dr. Ron Slaby in collaboration with Harvard Medical School. It is designed for middle-school students and follows a 12-step curriculum. The curriculum provides children with behavioral skills that can be used when they are in the roles of aggressors, victims, and bystanders. Each lesson is designed to be taught in 45-minute sessions, but can be customized for each classroom. The curriculum consists of role-playing activities, class discussions, small group work, and conflict resolution homework. The main goal of the curriculum is to teach students a four-step “Think First Model,” a cognitive approach to dealing with conflict non-violently. AVB has been identified as an effective violence prevention program by the U.S. Department of Justice and U.S. Department of Education .^30-31^

AVB was strongly evidence-based and fulfilled all of the search criteria. It was selected because of its affordability, pre-designed curriculum and materials, classroom, customizability, focus on the middle-school age range, and focus on social competencies and behavioral patterns related to youth violence. The AVB program also addresses the role of the bystander in violence, which was designated as an important and distinctive characteristic of the curriculum. In addition, AVB incorporated lessons that were identified as important for youth violence prevention through our discussion group. Each of the 12 sessions in AVB relies on peer-learning, such as small group work and role-playing skits, instead of teacher instruction. Within the 4-step “Think First Model” for responding to conflict, students develop long-term educational and career goals. Common beliefs about aggressive behavior, such as the ability to gain power through violence, are discussed in the context of the influences of the media. 

Investigators collaborated with the Superintendent of Metropolitan Nashville Public School (MNPS) to reach out to 15 middle schools that sought school violence prevention programs. One school in particular presented needs that most conflated with the goals of the AVB curriculum and was selected for our pilot intervention. AVB was then customized, according to requests by the pilot school, for the 6th-Grade health courses for the fall 2012 semester. Authors re-aligned the curriculum with the 70-minute courses taught by the school health counselor. The collaborators discussed the components of the AVB curriculum with the health counselor, including the use of a classroom projector, slides/transparencies, homework assignments, in-class role plays, activities, and skits.

**Pilot evaluation**

The fourth component of this study involved piloting AVB at the pilot school for the fall semester of 2012. The AVB curriculum was taught from September 2012 to December 2012. Investigators attended one class weekly in order to ensure the appropriate adoption and implementation of the curriculum.

122 students were divided into five classes, two of which were assigned to Group 1 and three of which were designated to Group 2. Each class was taught the AVB curriculum for ten weeks. A pre-test/post-test modeled after a test designed by Dr. Ron Slaby that was used in pilot evaluations in other studies was utilized. The pre-test was administered to the 71 students in Group 1. Group 2, which consisted of 51 students, only took the post-test. Their responses were used to determine if there was any pre-test bias, which is a change in participants’ answers due to previous knowledge of test questions. 

The pre-test/post-test consists of 32 questions with answers given on a Likert scale on the following subjects: (a) behaviors and histories related to violence, (b) beliefs about violence in the community, and (c) competencies to handle violent situations. Questions addressed all three roles of aggressor, victim, and bystander. All pre-/post-test questions consisted of one of two numerical Likert scale systems: 1 = “Never”, 2 = “Almost Never”, 3 = “Sometimes”, 4= “Often”, 5 = “Almost Always”, 6 = “Always”; and 1 = “Disagree Completely”, 2 = “Disagree A Lot”, 3 = “Disagree A Little”, 4= “Agree A Little”, 5 = “Agree A Lot”, 6 = “Agree Completely”. All answers remained anonymous and were collected and recorded into a secure Microsoft Excel spreadsheet. Following the program, qualitative feedback from educators and student participants were given verbally. Answers were recorded on paper. 

**Statistical Analysis**

For each question requiring a numerical answer, scores were totaled, averaged, and run through statistical package software SPSS. Responses from students who were learning English as a second language (ESL students) were excluded from the analysis. A non-parametric Mann-Whitney U Test was used to analyze pre-test and post-test differences in the average score on all numerical questions for Group 1 (α =.05). Differences in post-test scores between Group 1 and Group 2 were analyzed to measure pre-test bias. 

A decrease in average score for questions related to violent actions and behavior was considered an improvement in behavior. On twelve questions that addressed non-violent actions and behavior, an improvement was considered an increase in scores. These scores were averaged and inverted. For each question requiring a written answer in a “blank,” answers were recorded qualitatively. If an answer was illegible or not given, that answer was designated as “N/A” and not added to the average. Qualitative data was consolidated and reviewed for content. General themes and concepts that were provided by more the instructor or student body have been reported. 

## Results

**Quantitative **

From Group 1, 55 students took the pre-test and 71 students took the post-test. 16 of the students were not present on the day of the initial test. 

Table 1 shows the average scores for questions on behavior and histories related to violence. Students showed improvement in the majority of these questions following the program, with significant improvement in three of these questions (p<0.05). Students responded they went from sometimes getting hit or pushed by others to almost never getting hit or pushed. Students also reported that after the program they never got beaten up or threatened with guns by others (even as a joke), demonstrating a significant decrease in victimization (p<0.05).

**Table 1:** Behaviors and histories related to violence before and after program.

	Pre-test	Post-test	p for sig. improvement	p for sig. pre-test bias
How often do you:				
Call other people names?	2.85	2.97		
Get made fun of by others?	3.13	2.49		
Let a fight start without trying to stop it?	2.78	2.69		
Stick up for yourself without fighting?	3.65	3.55		
Get picked on by others?	2.71	2.45		
Hit or push others?	2.72	2.34		
**Get hit or pushed by others?**	**2.72**	**1.97**	**.027**	
Try to keep others from fighting?	3.44	3.24		
**Get threatened with guns by others (even as a joke?)**	**1.98**	**1.38**	**.010**	
Pick fights with others?	1.96	1.38		
**Get beaten up?**	**1.69**	**1.25**	**.045**	
Cheer when others fight?	2.33	2.03		
Try to break up a fight?	3.18	2.24		
At school, how often do your classmates:				
Threaten people with violence?	2.39	2.52		.029
Use physical violence?	2.14	2.32		.001

Values listed are mean responses rounded to nearest tenth based on Likert scale: 1= never; 2 = almost never; 3 = sometimes; 4 = often; 5 = almost always; 6 = always. p-value for sig.<.05. The shaded areas represent significant improvements after program.

Table 2 shows student’s beliefs on violence in the community before and after the program. Although none of the student responses showed significant improvement, the answers were trending towards improvement with students more likely to agree that they can discuss stopping school violence comfortably with friends and teachers.

**Table 2:** Beliefs on violence in the community before and after program.

	Pre-test	Post-test	p for sig. improvement	p for sig. pre-test bias
What are your beliefs on the following:				
I have the tools to handle violence at my school	2.99	3.42		
A school violence prevention would be helpful at my school	3.55	3.84		
I can discuss stopping school violence&gun violence comfortably with my friends/teachers	3.06	3.66		

Values listed are mean responses rounded to nearest tenth based on Likert scale: 1= disagree completely; 2 = disagree a little; 3 = disagree a little; 4 = agree a little; 5 = agree a lot; 6 = agree completely. p-value for sig. <.05.

Improvement was shown in students’ competencies to handle violent situations (Table 3). They were more likely to “size up the situation” in a bullying scenario, a thought process highlighted in the AVB curriculum as a tool to prevent violence before it occurs. They were also significantly more likely to have completed homework related to personal conflicts (p<.05).

**Table 3:** Competencies to handle violent situations before and after program.

	Pre-test	Post-test	p for sig. improvement	p for sig. pre-test bias
How many times do you do the following?				
“Size up the situation” in a bullying case?	**2.22**	**2.93**	**.005**	
“Think it through” in a bullying case?	2.98	3.24		
Take notes on a personal conflict or conflict at school?	1.92	2.24		
Do homework about personal conflicts?	**1.96**	**3.28**	**.001**	

Values listed are mean responses rounded to nearest tenth based on Likert scale: 1= never; 2 = almost never; 3 = sometimes; 4 = often; 5 = almost always; 6 = always. p-value for sig. <.05.

Comparing the average post-test responses for Groups 1 and 2 showed there was minimal overall pre-test bias. As demonstrated in Table 1, only three questions were shown to have significant pre-test bias. None of the questions with pre-test bias showed significant improvement in responses. 

**Qualitative **

The authors received verbal feedback from counselors, teachers, and administrators. Educators supported the AVB program and acknowledged that students were more aware of potentially violent behaviors. They believed that students had increased competencies to avoid violence. The school counselors engaged the AVB concepts, and encouraged a new role for students called the “non-violent problem solver.” 

After the post-test, the seventy-one students in Group 1 were asked to give verbal feedback about AVB curriculum. Positive feedback was given for the general subject matter about violence, role-playing, small group discussions, illustrated handouts, in-class slides, and the opportunity to share their opinions. Some students said the classes tended to be boring and long. Student suggestions for the class were more group-work, more relevant examples of violent situations, and more engaging activities.

## Discussion

The success of school-based violence prevention programs remains dependent upon appropriate development, implementation, and evaluation. The results of our study showed that students felt less victimized by their classmates both verbally and physically after learning the AVB curriculum. Although student responses to questions related to their roles as aggressors and bystanders did not show significant improvement, their responses trended toward improvement for most of these questions. The lack of significant data may have been due to our small sample size in our pilot. It may also be true that students are more easily able to recall situations in which they were victimized than situations in which they acted as the aggressor or in the passive role of bystander. 

Qualitative data also provided important information on acceptance of the program. Overall, the program received positive reviews from school administrators, teachers, and students. Student feedback showed that shorter sessions as described in the original AVB curriculum may be more engaging and appropriate for student attentiveness. 

Leff et al. recently designed a school-based violence prevention program for urban youth by holding discussion groups with community stakeholder groups, conducting literature reviews to draft a program, pilot testing the program in after-school centers, and engaging with community members in development and implementation of the project.^20^ Although we also held discussions with community stake-holders, we included participants that were formerly involved in youth violence instead of parents and community leaders in order to gain a peer perspective of youth violence. In addition Leff et al. utilized individual components of several programs they found through their literature review to develop a draft of a new 10-session youth program. Although this allowed for the development of a specialized program for their target community, this method depends on the assumption that modification and selection of individual components from a best practice program maintains the effectiveness of the program the way that it was originally designed. In our study, we utilized our review to select a single program for implementation while making modifications during classroom instruction specifically in order to enhance its effectiveness in our target population. 

In a study on the development and evaluation of school-based violence prevention programs, Farrell et al. discuss the importance of collaborating with experts in violence prevention in addition to informed stake-holders that have extensive knowledge of major problems within the target community.^17^ In our study, both the youth specialist who facilitated our discussion groups and the participants themselves provided insight into the behavioral and cognitive trends that need to be targeted in early adolescence. The “Think First Model” in the AVB curriculum was discovered to address these behaviors by providing students with the necessary cognitive thought processes to navigate through conflict, such as building long-term career goals. 

The implementation of the AVB program was also another important factor in the success of the AVB curriculum. In a study on strategies that improve replications of evidence-based programs, Fagan et al. describes the importance of support from key participants (such as school administrators and teachers) and continued involvement with program coordinators in order to meet the challenges that develop from introducing curriculums within the school system.^19^ During the implementation of AVB, we ensured that a strong partnership existed between our research team and school administrators. Due to effective communication and continued presence of our research team at the pilot school, we were able to adopt the AVB curriculum to fit appropriately within the set school schedule. 

**Limitations**

As a pilot study that was designed to determine the potential of a large-scale intervention, our sample size was relatively small and the results were analyzed with moderate rigor. Because the analysis was limited to self-reported pre-test/post-test score differences and qualitative feedback of a young population, the accuracy of our findings remains limited. In order to reduce the effect of self-report bias, we utilized a questionnaire that was previously validated by Slaby et al. in several studies implementing AVB in school curricula.^28^ In addition, the intervention was not assigned randomly to a school but was instead based on the input of the school district administrators. 

A control group was not assigned based on the decision of the school administrators to allow all students an equal opportunity to learn from the pilot program. Although pre-test bias was still considered, other confounding variables may have led to our observed differences. Student identification numbers were also not recorded at the request of school administrators, but would have allowed us to identify the sixteen students that were present for the post-test and not the pre-test. However, our method of doing a statistical analysis of an average of student scores allowed us to overcome this limitation to an extent. In order to ensure the adoptability of the AVB curriculum, the health counselor was allowed to make adjustments to the curriculum. Although this reduces the generalizability of the study, it also allowed us to evaluate and address the common problems that occur outside of a rigorous research study.^19^

Finally, our post-test only allowed us to identify student thoughts and behavior within a short duration after the AVB curriculum was taught and did not give evidence of long-term effectiveness. Although this analysis was beyond the scope of the short-term pilot study, it is being considered for a longer term, multi-school intervention.

## Conclusion

The success of Aggressors, Victims, and Bystanders at reducing violent behavior in a high-risk middle school in Nashville, Tennessee demonstrated its potential for expansion into a large-scale intervention. However, further research is needed to demonstrate the effectiveness of the program in the longer term. There is also a need to evaluate the program beyond self-reported student questionnaires. For example, data on the number of violent occurrences for which disciplinary action is given may provide greater evaluation of the program. We recommend our systematic review and discussion group model for successful selection of violence prevention programs in other locations.

The statistical results of this study and the qualitative feedback from educators and students are currently being incorporated to develop a more effective curriculum and study design for a large-scale impact evaluation study. Changes include removing test questions that showed significant pre-test bias, increasing the sample size, and creating a control group. Teachers will be advised by suggestions from educators in our pilot study on how to better adopt the program for Nashville middle school students, and will again be encouraged to adopt the program for their classroom.

In the fall of 2013, the revised curriculum will be implemented to eight or more middle schools in Nashville, Tennessee and will encompass the entire school from fifth to eighth grade. The large-scale impact will address the statistical limitations of the pilot while demonstrating the feasibility of introducing Aggressors, Victims, and Bystanders across all middle schools in Nashville. 
